# Microalbuminuria in diabetes mellitus: Association with age, sex, weight, and creatinine clearance

**DOI:** 10.4103/0971-4065.53322

**Published:** 2009-04

**Authors:** N. K. Chowta, P. Pant, M. N. Chowta

**Affiliations:** Departments of Medicine and Pharmacology, Kasturba Medical College, Mangalore, India

**Keywords:** Microalbuminuria, diabetes mellitus, blood pressure, obesity

## Abstract

Studies in the Western literature show a linear relationship between degree of microalbuminuria and body mass index (BMI), blood pressure, and duration of diabetes. This study was aimed to determine the correlation of microalbuminuria with age, sex, duration of diabetes, BMI, and creatinine clearance in type-2 diabetics in Indian population. One hundred patients (59 males and 41 females) with type-2 diabetes mellitus of duration six months or more and negative for albumin in urine by albustic method were included in the study. Detailed clinical history was taken followed by a thorough physical examination that included neurological examination in the selected patients. Micral test was used for estimation of microalbuminuria. Overall prevalence of microalbuminuria in the present study was 37%. Among the patients with microalbuminuria, 20 were males and 17 were females. Pearson correlation of microalbuminuria with age showed statistically significant linear relationship. Gender-wise correlation analysis of microalbuminuria failed to show any statistical significance. Correlation of microalbuminuria with BMI was also not significant (*r* = 0.063, *P* > 0.05). Creatinine clearance negatively correlated with microalbuminuria, but this was statistically insignificant. There was a statistically significant correlation of microalbuminuria with duration of diabetes. Prevalence of microalbuminuria is around 37% in type-2 diabetes mellitus. Incidence of microalbuminuria increases with age as well as with increased duration of diabetes mellitus. There is no effect of BMI and sex on the prevalence of microalbuminuria.

## Introduction

The term microalbuminuria is defined by a urinary albumin excretion (UAE) rate higher than normal but lower than 200 *μ*g/min, the lowest detection limit of proteinuria as measured by standard laboratory methods[1,2] in the absence of urinary tract infection and acute illness including myocardial infarction.[3] Albumin excretion in healthy individuals ranges from 1.5–20 *μ*g/ min.[4] The presence of microalbuminuria precedes the development of overt diabetic nephropathy by 10–14 years. It is at this stage that one can hope to reverse diabetic nephropathy or prevent its progression. Therapeutic interventions which reverse microalbuminuria include intensified glycemic control, use of ACE inhibitors, etc. A diagnosis of microalbuminuria can be made by measuring its excretion rate during 24 hours or in an overnight urine collection, or by measuring albumin/creatinine ratio or albumin concentration in the morning or a random urine sample. Determination of UAE in the morning urine sample constitutes the ideal test for screening, and overnight urine collection might be the best choice for monitoring microalbuminuria.[1] In type-2 diabetes mellitus prevalence of microalbuminuria ranges from 8–47%.[6,7] Microalbuminuria is the strong predictor of diabetic nephropathy, which is the main cause of morbidity and mortality in patients with diabetes mellitus. Microalbuminuria is also characterized by increased prevalence of arterial hypertension, proliferative retinopathy, and peripheral neuropathy. Studies in the Western literature have documented the linear relationship of degree of microalbuminuria with body mass index (BMI), blood pressure, and duration of diabetes. Gender correlation of microalbuminuria was not seen in type-2 diabetes mellitus.[8,9] This study was aimed to determine the prevalence of microalbuminuria in type-2 diabetic patients and to evaluate the relation between microalbuminuria and age, sex, duration of diabetes, body mass index, and creatinine clearance.

## Materials and Methods

This cross-sectional prospective observational study was carried out between July 2004 and June 2005, to investigate the correlation between microalbuminuria and assumed risk factors. Study was approved by the institutional ethics committee and written informed consent was taken from all the patients. Patients visiting our outpatient unit were screened for eligibility into the study. Hundred patients with type-2 diabetes mellitus of duration six months or more and negative for albumin in urine by albustic method were included in the study. Patients with overt albuminuria (>350 mg/day), congestive cardiac failure, urinary tract infection, pregnant patients, patients confined to bed for more than two weeks, and patients on ACE inhibitors for hypertension were excluded from the study. Other causes for microalbuminuria like heavy metal poisoning, connective tissue disorders, and chronic NSAIDs use were also ruled out in the selected patients. The selected patients were studied in detail with history and physical examination, including detailed neurological examination. Body mass index (BMI) was calculated from the height and weight measurements of the patients. Routine investigations including serum creatinine were done in all the selected patients. Creatinine clearance was calculated based on MDRD formula.

In the present study, micral test was used for estimation of microalbuminuria (Boehringer Mannheim, West Germany). The micral test is a test-strip method in which the color reaction is mediated by an antibody-bound enzyme.[1] This method has shown good correlations with radioimmunoassay and can be readily used for screening.

All patients were afebrile during the collection of urine. Urine was first tested for albumin by albustick method (Combur test). Only those patients who were negative for albumin in urine by albustic method were included in this study. First morning mid-stream urine sample was collected in sterile container. Test strip was immersed in urine such that the fluid level was between two black bars. Strip was withdrawn after five seconds. Strip was placed horizontally across the urine vessel and the color change in test zone was compared with color scale after one minute. Sensitivity of the kit is 0.4 ng/ml and the measuring range is 0.8–10 ng/ ml. Microalbuminuria was graded as mild (20–50 mg/L), moderate (50–100 mg/L), or severe (100–300 mg/L) depending on the color change in the strip. Test was repeated twice for selecting the patients into the study.

### Statistical analysis

Data collected were analyzed by student's ‘*t*’ test or chi-square test as appropriate. Pearson correlation test was used to analyze the correlation of microalbuminuria with independent variables like age, sex, BMI, duration of diabetes, and creatinine clearance. Probability (P) value less than 0.05 was regarded as statistically significant.

## Results

A total of 100 patients, 59 males and 41 females, were included in the study. Overall prevalence of microalbuminuria in the present study was 37%. Among the patients with microalbuminuria, 20 (54.05%) were males and 17 (45.95%) were females. Among 37 microalbuminuric patients, 9 patients had mild albuminuria, 18 had moderate albuminuria, and 10 had severe albuminuria. Baseline characteristics of the patients are shown in Table 1. Age of patients at diagnosis ranged between 30–70 years. Mean age at onset of diabetes mellitus in microalbuminuric patients was 51.7 ± 9.8 years and in normoalbuminuric patients it was 46 ± 11.6 years. The difference between the two groups was statistically significant. Gender-wise comparisons of baseline characteristics are shown in Table 2. There was statistically significant difference in creatinine clearance between males and females. Creatinine clearance was much lesser in females. Pearson correlation of microalbuminuria with age showed significant linear relationship (*r* = 0.529, *P* < 0.001). Gender-wise correlation analysis of microalbuminuria was not significant [Table 3]. Eleven patients had BMI >30 kg/m^2^, among them four had microalbuminuria (10.8%) and seven had normoalbuminuric (11.1%). Mean BMI of microalbuminuric patients was 22.4 ± 6.9 kg/m^2^ and for normoalbuminuric patients it was 21.6 ± 4.2 kg/m^2^. The difference between the groups was not statistically significant. Pearson correlation analysis also did not show any significance for microalbuminuria and BMI (*r* = 0.063, *P* > 0.05). Creatinine clearance negatively correlated with microalbuminuria, though statistically insignificant (*r* = −0.158, *P* > 0.05). Maximum number of patients (54) had duration of diabetes between six months and five years [Table 4]. Among these, four (7.4%) had microalbuminuria. Twenty four patients had duration of diabetes between five and ten years. Among them 12 (50%) had microalbuminuria. Eleven patients were with duration of diabetes between 10 and 15 years, among them 10 (90.9%) were positive for microalbuminuria. Remaining eleven patients had duration of diabetes more than 15 years, all of them were positive for microalbuminuria (100%). Mean duration of diabetes in microalbuminuric patients was 10.7 ± 5.0 years while in normoalbuminuric patients it was 3.2 ± 2.0 years, which was statistically highly significant. Pearson correlation analysis showed statistically significant correlation of microalbuminuria with duration of diabetes (*r* = 0.839, *P* < 0.0001, Table 3). Among the 100 patients, 84 were only on oral hypoglycemic agents, four were on insulin, and 12 were on both insulin and oral hypoglycemic agents. Among microalbuminuric patients 19 had severe diabetes, 14 had moderate diabetes, and 4 had mild diabetes. Among the normalbuminuric patients, nine had severe diabetes, 41 had moderate diabetes, and 13 had mild diabetes. Average fasting blood sugar was 218 ± 52.5 mg/dl in microalbuminuric patients which was higher than normoalbuminuric patients (177.5 ± 28.9 mg/dl). Relationship between severity of diabetes and microalbuminuria was significant.

**Table 1:** Baseline characteristics of the patients

Variable	All patients	Range	Microalbuminuric patients (*n* = 37)	Normoalbuminuric patients (*n* 63)	*P* value
Sex (M/F)	59/41		20/17	39/24	
Mean age (years)	54.18 ± 13.73	30–85	62.49 ± 12.14	49.3 ± 12.25	<0.001
Mean age at onset of diabetes (years)	48.6 ± 10.5	28–70	51.7 ± 9.8	46 ± 11.6	<0.001
BMI (kg/m^2^)	22.09 ± 4.99	11.6–38.2	22.88 ± 6.06	21.64 ± 4.23	0.23
Serum creatinine (mg/dl)	0.97 ± 0.17	0.6–1.3	0.98 ± 0.16	0.97 ± 0.18	0.61
Creatinine clearance (ml/min/m^2^)	80.35 ± 21.81	43–166	75.43 ± 20.57	83.24 ± 22.16	0.08
Duration of diabetes (years)	5.97 ± 4.98	1–20	10.66 ± 5.02	3.21 ± 2.01	<0.001

All values are expressed as mean ± SD; Student's ‘*t*’ test

**Table 2:** Gender-wise comparison of baseline characteristics

Variable	Males (*n* = 59)	Females (*n* = 41)	*P* value
Mean age (years)	62.97 ± 12.95	55.93 ± 14.78	0.29
BMI (kg/m^2^)	21.66 ± 4.77	22.73 ± 5.29	0.29
Serum creatinine (mg/dl)	0.97 ± 0.18	0.97 ± 0.17	0.96
Creatinine clearance (ml/min/m^2^)	90.37 ± 20.65	65.93 ± 14.06	<0.001
Duration of diabetes (years)	5.68 ± 5.08	6.39 ± 4.86	0.48

All values are expressed as mean ± SD; Student's ‘*t*’ test

**Table 3:** Correlation of microalbuminuria with independent variables

Variables	Mean ± SD	Correlation coefficient (*r*)
Age (years)	54.18 ± 13.73	0.529*
Sex (M/F)	59/61	0.062
BMI (kg/m^2^)	22.09 ± 4.99	0.063
Duration of diabetes (years)	5.97 ± 4.98	0.839**
Creatinine clearance (mg/dl)	80.35 ± 21.81	−0.158

Pearson correlation;

* *P* < 0.001

** *P* < 0.0001

**Table 4:** Prevalence of microalbuminuria in relation to duration of diabetes mellitus

Duration of diabetes (years)	No. of microalbuminuric patients (%)	No. of normoalbuminuric patients (%)	Total (*n*)
1-5	4 (7.4)**	50 (92.6)	54
5-10	12 (50)	12 (50)	24
10-15	10 (90.9)*	1 (9.1)	11
>15	11 (100)**	0	11

Chi-square test;

* *P* < 0.01

** *P* < 0.001

## Discussion

This cross-sectional study presents data on prevalence and associations of microalbuminuria with various parameters in type-2 diabetes mellitus. Present study has shown prevalence of microalbuminuria at 37%, which is much higher when compared to the study by Ghai *et al*, where prevalence was reported at 25%.[5] Higher prevalence in the present study may be due to the fact that most of the patients were on irregular treatment with poor glycemic control and also may be due to the small sample size. Method of estimation of microalbuminuria as well as ethnical differences would have also played a role in giving higher prevalence in the present study. The level of glycemic control seems to be the strongest factor influencing transition from normoalbuminuria to microalbuminuria.

Present study has shown statically significant linear relationship of degree of albuminuria with age. Earlier studies have also shown positive correlation of microalbuminuria with age of the patients.[8,9] Our study has not shown gender-wise correlation of microalbuminuria, which is in contrast to the previous studies that have reported male dominance in the prevalence of microalbuminuria. As reported in many studies, our study failed to show any correlation between BMI and microalbuminuria.[8,9] This may be due to the confounding variables like duration of diabetes and glycemic control that would have played a major role in the occurrence of microalbuminuria.

Creatinine clearance has shown slight negative correlation with microalbuminuria in the present study, though statistically insignificant. Serum creatinine and creatinine clearance was within normal range in all the patients. Diabetic nephropathy can conveniently be categorized into different stages with respect to renal hemodynamics, systemic blood pressure, urinary findings, and susceptibility to therapeutic interventions. In the initial renal hyperperfusion stage, glomerular filtration is elevated with absent albuminuria. In the second stage (clinical latency) glomerular filtration will be high normal with absent albuminuria. Next stage is incipient nephropathy, wherein glomerular filtration will be normal with presence of microalbuminuria. It usually appears 5–15 years after the diagnosis of diabetes mellitus. In the subsequent stage, glomerular filtration decreases with appearance of macroproteinuria and clinical manifestations of nephropathy. Finally ends up in endstage renal disease with massive albuminuria and diminished glomerular filtration.[10] Hence microalbuminuria may not be associated with abnormal serum creatinine or creatinine clearance, but can be an important warning signal which if ignored can result in irreversible renal damage.

Present study has shown positive correlation of microalbuminuria with duration of diabetes mellitus which is in accordance with many previous reports. Duration of diabetes has significant contribution for the development microalbuminuria by prolonged exposure to hyperglycemia-induced advanced glycosylation end products accumulations. Control of diabetes with regular treatment also plays a significant role in the development of diabetic nephropathy.[11–13]

Limitations of the present study must also be considered. As our study was not based on the general population, selection bias might have affected the outcome of the study. Larger sample size in general population may be required to confirm the results of the present study.

In conclusion, our study has found higher prevalence of microalbuminuria (37%) in type-2 diabetes mellitus, which is the predictor of later development of diabetic nephropathy. Incidence of microalbuminuria increases with age as well as with increased duration of diabetes mellitus. There is no effect of BMI and sex on the prevalence of microalbuminuria in type-2 diabetes mellitus. Results of our study confirm and extend the previous observations in small selected groups of patients with type-2 diabetes mellitus. Creatinine clearance will be within normal range in microalbuminuric patients. But the presence of microalbuminuria alerts the physician to prevent further renal damage by timely administration of ACE inhibitors and correction of risk factors. Urinary excretion of albumin should be monitored routinely in patients with diabetes mellitus.
